# Immunotherapeutic Implications of Toll-like Receptors Activation in Tumor Microenvironment

**DOI:** 10.3390/pharmaceutics14112285

**Published:** 2022-10-25

**Authors:** Run Zheng, Jian Ma

**Affiliations:** 1 NHC Key Laboratory of Carcinogenesis, Hunan Cancer Hospital and the Affiliated Cancer Hospital of Xiangya School of Medicine, Central South University, Changsha 410013, China; 2 Cancer Research Institute and School of Basic Medical Science, Central South University, Changsha 410078, China; 3 Key Laboratory of Carcinogenesis and Cancer Invasion of the Chinese Ministry of Education, Hunan Key Laboratory of Nonresolving Inflammation and Cancer, Changsha 410078, China

**Keywords:** toll-like receptors, tumor microenvironment, cancer immunotherapy, vaccine adjuvant, combination therapy

## Abstract

Toll-like receptors (TLRs) play an important role between innate and adaptive immunity as one of the pattern recognition receptors (PRRs). Both immune cells and tumor cells express TLRs, and the same TLR molecule is expressed in different cells with different roles. TLR activation in the tumor microenvironment mostly has a dual role in tumor progression during chronic inflammation. Clinically, the therapeutic efficacy of most cancer immunotherapy strategies is restricted by the suppressive immune infiltrative environment within the tumor. Therefore, activation of TLRs in innate immune cells has the potential to eradicate tumors lacking T-cell infiltration. TLR agonists have served as important immunomodulators of cancer immunotherapy through immune responses and reprogramming the tumor suppressive microenvironment. Meanwhile, considering the complex interaction of TLRs with the tumor microenvironment, a combined approach of cancer immunotherapy and nanotechnology has been adopted to improve cancer immunotherapy not only by combining multiple drug combinations, but also by targeting the tumor microenvironment using nanoparticles. Many clinical trials are underway to improve antitumor activity through combination with other immunotherapies. In this review, we provide a comprehensive and detailed overview of the immunotherapeutic implications of TLRs activation in tumor microenvironment, highlighting its great potential to be an important tool for cancer immunotherapy.

## 1. Introduction

Toll-like receptors (TLRs) are mainly expressed in immune cells and are involved in the innate immune response triggered by the recognition of multiple pathogen components, as well as in the induction and regulation of adaptive immune responses [1,2]. TLRs are also expressed in some tumor cells and act as a double-edged sword, because uncontrolled TLR signaling provides a microenvironment favoring tumor cell proliferation and evasion of from immune responses [3,4]. TLR signaling can promote chronic inflammation-induced tumorigenesis [5], and chronic inflammation is a key feature of the tumor microenvironment (TME), which not only stimulates tumor cell proliferation and survival, but also suppresses anti-tumor immunity.

T-cell activation is impaired in cancer patients due to immunosuppression, loss of tumor antigen expression, and antigen-presenting cell dysfunction. The search for therapeutic modalities that promote T-cell infiltration into human tumors and achieve optimal clinical efficacy requires additional combination regimens. In addition, most tumors may exhibit drug resistance. Methods to resensitize drug-resistant tumors to immunotherapy include direct and indirect modulation of immunogenicity. A rational combination of microenvironment-targeted therapies and immune checkpoint inhibitors or cellular therapies will be the next generation of immune-based cancer therapies [6].

TLR agonist treatment enhances the activity of anti-cancer effector cells while blocking the activity of immunosuppressive cells. It also affects the recruitment of immune cells to tumors. Based on these unique properties, TLR agonists can be used as monotherapy or combination modality strategies for cancer treatment [7,8].

## 2. Activation of Toll-like Receptors

### 2.1. Toll-like Receptors

TLRs are transmembrane pattern recognition receptors and belong to one of several pattern recognition receptors (PRRs), and others include RIG-I-like receptors, NOD-like receptors and C-type lectin receptors. Their activation occurs early in the inflammatory cascade response [9]. They play an important role in the activation of innate and adaptive immunity by recognizing pathogen-associated molecular patterns (PAMPs) such as unmethylated cytosine guanosine dinucleotide (CpG), single-stranded RNA (ssRNA), lipoproteins, lipopolysaccharide (LPS) and flagellin for defense against pathogen invasion [10]. Some TLRs are also involved in the recognition of damage-associated molecular patterns (DAMPs) as receptors for inflammation and injury, which may be associated with cancer development [11].

TLR’s function in innate immunity was firstly reported in Drosophila that Drosophila mutants in the Toll gene are highly susceptible to fungal infection. Subsequently, ten TLR family members were identified in humans, while thirteen TLR family members were found in mice. Among them, TLR1, TLR2, TLR5 and TLR6 are located on the cell membrane, TLR3, TLR7, TLR8 and TLR9 are located on the endosomal membrane, while TLR4 is distributed on both cell and endosomal membranes [10]. They are expressed in various immune cells (dendritic cells, macrophages, T cell subsets and B cells) and non-immune cells (epithelial cells and fibroblasts) [12].

### 2.2. TLR-Activated Pathways

**Figure 1:**
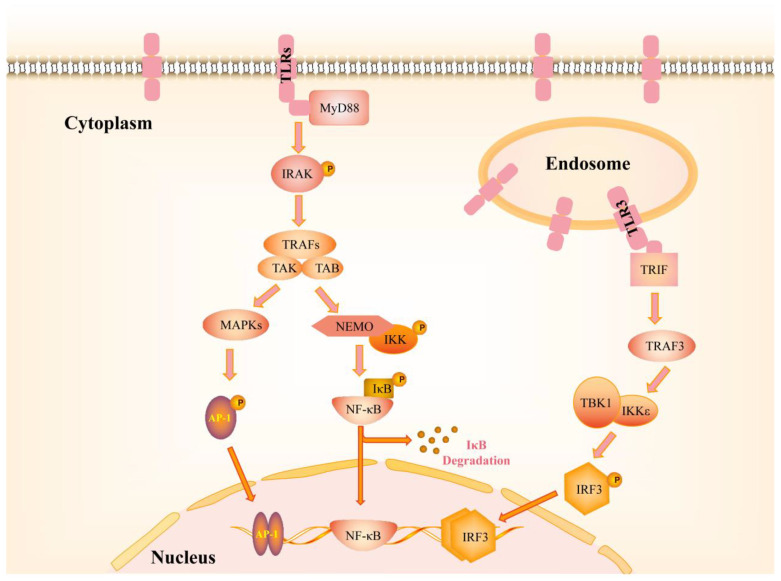
TLR-mediated intracellular signaling pathways. All of TLRs, except TLR3, initiate MyD88-dependent signaling pathways, activating NF-κB and MAPKs. TLR3 is located in the endosomal membrane and initiates the TRIF-dependent signaling pathway, mediating the production of pro-inflammatory cytokines and type I interferon. MyD88: myeloid differentiation primary response gene (88); IRAK: interleukin-1 receptor-associated kinase; TRAFs: TNF receptor-associated factors; MAPKs: mitogen-activated protein kinases; AP-1: activator protein 1; TRIF: TIR domain-containing adaptor inducing IFNβ; TBK1: TANK-binding kinase 1; IRF3: interferon regulatory factor 3.

TLRs consist of an extracellular leucine-rich repeat (LRR) recognition domain and an intracellular Toll/IL-1 receptor (TIR) domain that transmits downstream signals. Activation of TLRs proceeds by dimerization of TLRs to form TIR-TIR structures that recruit intracellular adaptor proteins that also contain TIR domains to activate NF-κB, p38 and JNK signaling transduction [13]. TLR-activated pathways include the MyD88-dependent pathway and the TRAF-dependent pathway. Upon TLR recognition of ligands, members of the TLRs family other than TLR3 recruit the adapters MyD88, Mal, IRAKs, or TRAFs, thereby initiating the MyD88-dependent pathway [14]. MyD88 recruits IL-1R-associated kinases (IRAKs) that are phosphorylated to bind to TRAF (tumor necrosis factor receptor-associated factor) which then interacts with the ubiquitin-conjugating enzymes and becomes polyubiquitinated. Activated TRAFs combine with TGF-β-activated kinase (TAK) and TAK1-binding protein (TAB) to phosphorylate IκB kinase (IKK). This activated IKK phosphorylates IκB, an inhibitor of NF-κB, and promotes its degradation, thereby activating NF-κB transcription factors and mitogen-activated protein kinases (MAPKs). Consequently, the nuclear translocation of NF-κB and AP-1 induces the production of inflammatory cytokines and activates the transcription of innate immune effector genes [15,16]. In the TRIF-dependent pathway, TLR3 signaling is completely dependent on the adaptor protein TIR domain-containing adaptor inducing IFN-β (TRIF), also named as Toll-IL-1 receptor (TIR) domain-containing adaptor molecule-1 (TICAM-1). TRIF consists of a proline-rich N-terminal region, a TIR domain and a C-terminal region. Among them, the TIR domain of TRIF interacts with the TIR domain of TLR3, and the N-terminal region is essential for further phosphorylation and activation of interferon regulatory factor 3 (IRF3) by recruiting TANK-binding kinase 1 (TBK1) and the inhibitor of nuclear factor κB kinase ε (IKKε, also known as IKK ε). Finally, IRF3 activation mediates the production of pro-inflammatory cytokines and type I interferon (IFN) [14] (Figure 1).

Dysregulation of TLR signaling is associated with many diseases, including cancer [17], infectious diseases (viral, bacterial, parasitic) [18], autoimmune diseases [19], gut diseases (microbial, chronic inflammation) [20], nervous system diseases (addiction, Alzheimer’s disease, glioma) [21], asthma [22], and immunodeficiency diseases [23]. Manipulation of TLR signaling is designed to block their activity in inflammatory diseases and enhance their signaling against cancer. TLR agonists have shown great potential as antimicrobial agents and vaccine adjuvants, while TLR antagonists are being developed as agents and drugs to suppress immune responses [21]. High expression of TLRs in antigen-presenting cells has the ability to induce antitumor mediators (e.g., type I interferon), thus, tolerance could be converted into antitumor responses by using TLR agonists in tumor therapy [11].

## 3. Tumor Microenvironment and TLRs Activation

### 3.1. Tumor Microenvironment and Cancer Development

The close interaction between tumor cells and stromal cells forms the tumor microenvironment (TME). These stromal cells include various immune cells, cancer-associated fibroblasts and endothelial cells [24]. In addition, a dynamic network of soluble factors, cytokines, chemokines, growth factors and adhesion molecules links cellular interactions to create the TME. The TME network stimulates extracellular matrix remodeling, expansion of abnormal vascular and lymphatic networks, and cell migration in the tumor mass, participating in tumor development [25]. Cellular competition exists in the TME, and cancer cells that survive chemotherapy may accumulate additional mutations that allow them to regenerate to more aggressive tumors [26].

Tumor immune microenvironment (TIME) mainly refers to the composition of immune cells and the characteristics of inflammatory response in tumors. The development of resolution technology has revealed the heterogeneity in immune composition, spatial distribution and function within tumors, which has laid the foundation for the classification of TIME. TIME, in which cytotoxic lymphocytes are distributed at the tumor margin with relatively little infiltration in the tumor core, is called infiltrative exclusion (I-E) TIME. (I-E) TIME is less immunogenic and is referred to as a “cold” tumor, an immune state in which adaptive immunity cannot be activated. In contrast, infiltrative inflammatory (I-I) TIMEs are considered immunologically “hot” tumor, characterized by a high infiltration of cytotoxic T lymphocytes expressing PD-1 and leukocytes and tumor cells expressing the immunosuppressive PD-1 ligand, PD-L1 [17]. Cancer progression is influenced by the complex interactions between tumor cells and host immune responses within the tumor microenvironment. The efficiency of immunotherapy is dependent on the type of TIME [27]. The TME and the initial tumor cells evolve together during tumorigenesis. During tumor progression, many of the “hallmarks of cancer” are associated with the TME, including the ability to induce proliferation and inhibit apoptosis; to induce angiogenesis and avoid hypoxia; to suppress the immune system and evade immune detection; and to activate immune cells to support invasion and metastasis [28].

### 3.2. Tumor Microenvironment and Cancer Immunotherapy

Cancer immunotherapies can be broadly classified into five categories: vaccine immunotherapy, cytokine immunotherapy, immune checkpoint blockade immunotherapy, adoptive cell transfer immunotherapy, and small molecule immunotherapy. Immune checkpoint inhibitors, such as antibodies targeting cytotoxic T-lymphocyte-associated protein 4 (CTLA-4) and programmed cell death protein 1/programmed cell death ligand 1 (PD-1/PD-L1) have been used in advanced melanoma, Merkel cell carcinoma, non-small cell lung cancer, cutaneous squamous cell carcinoma, urothelial cancer, renal cancer, refractory Hodgkin lymphoma, hepatocellular carcinoma, gastric cancer, and triple-negative breast cancer, etc. The success of therapeutic suppression with immune checkpoint inhibitors has been limited to “hot” tumors characterized by preexisting T-cell infiltration, while “cold” tumors lacking T-cell infiltration have not achieved lasting benefit. The inability of “cold” tumors to produce spontaneous immune infiltration is due to the generation of an immunosuppressive TME [29]. T cells engineered to express tumor-specific chimeric antigen receptors (CAR-T) have achieved remarkable success in the treatment of hematologic malignancies, but the presence of immunosuppression, antigen escape, and tumor defense mechanisms in the solid TME makes the application of CAR-T therapy in the treatment of solid tumors a significant limitation. Chimeric antigen receptors (CARs) are synthetic receptors that allow T cells to recognize tumor-associated antigens (TAAs) in a manner that is independent of the major histocompatibility complex (MHC). To enhance therapeutic efficacy in this immunosuppressive environment, CAR-T cells are engineered to induce TME remodeling to enhance endogenous antitumor immune responses, including remodeling of tumor cell composition and phenotype, and secretion of cytokines or soluble factors [30]. Alternatively, the immunosuppressive environment that disrupts the effect of CAR-T therapy can be counteracted by the addition of checkpoint blockade drugs [31].

### 3.3. Expression and Activation of TLRs in Tumor Microenvironment

TLRs are mainly expressed in human immune cells, however, they are also expressed in many tumor cell lines or tumor tissues, especially in cancers of epithelial origin, and the type of TLRs expressed on different tumor cells varies and is related to tumor progression [32].

In the TME, TLRs are expressed on both immune cells and tumor cells and play a dual role, triggering both anti-tumor (innate and adaptive immunity) and pro-tumor (cell proliferation, migration, invasion, and cancer stem cell maintenance) responses [33,34]. DAMPs originating from damaged normal epithelial cells and necrotic cancer cells are present at significant levels in the TME, and their stimulation of specific TLRs can promote chronic inflammation. DAMPs include heat shock proteins (HSP 60, 70), ATP and uric acid, the calcium regulatory protein S100 family, the nuclear protein high mobility group box 1 (HMGB1), and nucleic acids. DAMP-induced chronic inflammation in the TME leads to an increase in immunosuppressive populations, such as M2 macrophages, myeloid-derived suppressor cells (MDSC) cells and regulatory T (Treg) cells. During chronic inflammation in the TME, TLRs play an active role in tumor progression [35,36,37,38]. When stimulated by endogenous molecules released from cancer-killed cells, TLRs activate NF-κB-mediated inflammatory responses. NF-κB-mediated inflammatory responses increase cancer cell stemness, and cancer stem cells constitutively exhibit higher NF-κB activation, which in turn increases tumor stemness, creating a positive feedback loop expanding cancer stem cell populations in tumors [39]. Activation of TLRs in tumor cells not only promotes tumor cell proliferation and anti-apoptosis, but also promotes tumor cell invasion and metastasis through regulation of metalloproteinases and integrins. In addition, activation of TLR signaling in tumor cells induces the synthesis of pro-inflammatory factors and immunosuppressive molecules that enhance the resistance of tumor cells to cytotoxic lymphocyte attack and lead to immune evasion [40].

**Figure 2:**
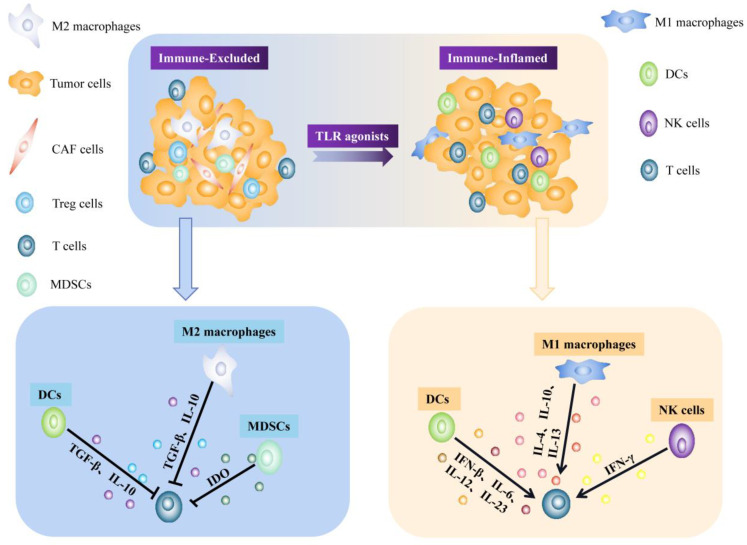
Reprogramming of the immunosuppressive TME by TLR agonists. Immune-Excluded (I-E) TIMEs are characterized by the exclusion of T cells from the tumor core, whereas MDSCs and Treg cells can suppress antitumor immune responses and promote malignant cell growth. And many cancers typically exhibit an immunosuppressive M2-like phenotype, in an anti-inflammatory state. TLR agonists treatment can transform the I-E TIME into an immune-inflamed (I-I) TIME. In the process of this transformation, TLRs activation is able to induce macrophage polarization toward the M1 type. And the cytokine profiles of dendritic cells (IL-6, IL-12, IFN-β and IL-23) and NK cells (IFN-γ) induced by individual TLR agonists enhance T-cell infiltration and alter the suppressive function of Treg cells.

TLR signaling also mediates the interaction of cellular energy metabolism molecules of tumor cells and tumor-infiltrating immune cells in the TME. Among them, tumor-derived metabolites maintain an effective tumor suppressive microenvironment [41]. Ubiquitination in TLR-dependent signaling pathways also regulates TME. Dysregulation of ubiquitination affects tumor metabolism, TIME, and cancer stem cells in tumorigenesis [42]. For example, Fcγ receptor IIb (FcγRIIb) is an inhibitory FcγR that is expressed on monocytes and macrophages. TLR4 ligands downregulate FcγRIIb through E3 ubiquitin ligase (MARCH3) mediated protein ubiquitination, which in turn affects the TIME [43] (Figure 2).

(1) Dendritic cells

Dendritic cells (DCs) act as an important anti-tumor component, though they are only a small part of the TME, because their ability to promote T-cell immunity and immunotherapeutic responses is very powerful [44]. Normally, plasmacytoid dendritic cells (pDCs) are capable of producing large amounts of type I interferon (IFN-α) and promote innate and adaptive immune responses. pDC can also act as antigen, presenting cells to regulate immune responses to various antigens [45,46]. However, in cancer, pDC exhibit an impaired response to TLR7/9 activation and reduced IFN-α production, contributing to the establishment of an immunosuppressive TME.

TLR signaling induces DC differentiation and affects its interaction with T cell. TLR ligands stimulate transcription of cytokines and costimulatory factors in DC, and TLR activation induces a series of cell biological changes in DC, including increased endocytic activity induced by the membrane vesicle system, regulation of the cytoskeleton and influence on their antigen presentation through protein translation and degradation mechanisms, further enhancing the quality, quantity and detectability of peptide-MHC complex formation in mature DC [47]. Signaling downstream of TLR directs the maturation of DC. Upon TLR activation, DC metabolism shifts from mitochondrial OXPHOS driven by β-oxidation of lipids to aerobic glycolytic metabolism, similar to Warburg metabolism in cancer cells. This metabolism is dependent on the phosphatidylinositol 3′-kinase (P13K)/Akt pathway, antagonized by adenosine monophosphate (AMP)-activated protein kinase (AMPK) and the anti-inflammatory cytokine IL-10, and is required for DC maturation [48]. In addition, vitro experiments have shown that mature DCs in a mixture containing IFN-γ and three TLR ligands including Resiquimod (R848), poly I:C and LPS were able to effectively stimulate TCR-transduced T cells by enhancing the production of IL-12p70 [49].

CD103^+^ cDC1 and CD11b^+^ cDC2 are the only TLR3-expressing groups at tumor sites. DCs produce the Th1-type cytokines IFN-β and IL-12 via the TLR3-TICAM-1 pathway, which contributes to the induction of anti-tumor immunity in functional cytotoxic T lymphocyte. Thus, tumor-specific cytotoxic T lymphocytes can be generated by targeting TLR3 signaling in DCs [50,51]. Comparative analysis of cytokine (IL-4, IL-6, IL-10, IL-12, and IL-23) profiles in DCs induced by single TLR agonist and multiple TLR agonists demonstrated synergistic effects among various TLR agonists on promoting cytokine production. Combined with the analysis of other cellular signaling pathways, the p38 MAPK and ERK signaling pathways were involved in IL-TLR-induced production of 12p70 and IL-12p40 in DCs; while the JNK pathway had a negative regulatory effect on cytokine production in DCs stimulated by certain TLR agonists; NF-κB and P13K acted as positive regulators when TLR signaling in DCs promotes cytokine production [52].

(2) Natural killer cells

Natural killer (NK) cells are important innate cytotoxic lymphocytes with the ability to rapidly and efficiently recognize and kill tumor cells. NK cells directly regulate T-cell activity through the production of IFN-γ and also shape the DC response to cancer through the induction of DC maturation and the recruitment of DC in the TME. NK cells are also regulated by many soluble cytokines in the TME. Cytokines including IL-2, IL-12, IL-15, IL-18 and IL-21 enhance NK cell activation, survival, proliferation and maturation upon binding to their cognate receptors. Strategies currently in preclinical and clinical development include adoptive transfer therapy, recruitment of NK cells to the TME, blockade of inhibitory receptors that limit NK cell function, and therapeutic modulation of the TME to enhance antitumor NK cell function [53].

The results of the adoptive transfer of autologous or allogeneic activated NK cells for the treatment of hematologic malignancies are encouraging, and the use of CAR-modified NK cells (CAR-NK) may be safer than CAR-T cell therapy. However, solid tumor is more difficult because NK cells are difficult to transport and penetrate to the tumor site and the TME impairs NK cell function. NK cell proliferation and antitumor activity are inhibited by the secretion of various immunosuppressive factors by tumor cells, including prostaglandin E2, indoleamine 2,3-dioxygenase (IDO), IL-10, TGF-β and vascular endothelial growth factor (VEGF) [54].

TLRs in NK cells induce innate immune responses against bacterial and viral infections by inducing NK cytotoxicity and cytokine production [55]. NK cells express distinct endosomal TLRs triggered by viral and bacterial-derived RNA and DNA. Four endosomal TLRs (TLR3, TLR7/8 and TLR9) are expressed homogeneously on CD56^bright^CD16^−^ and CD56^dim^CD16^+^ NK cell subsets, but TLR7/8 (R848), TLR3 (Poly I:C) and TLR9 (ODN2395) ligands promote NK cell function only in the presence of cytokines, including interleukins (IL-2, IL-12, IL-15 and IL-18). Among them, R848 activates CD56^bright^CD16^−^ NK cells mainly by promoting cell proliferation, cytokine production and cytotoxic activity. R848, which normally activates both TLR7 and TLR8 on DCs, macrophages and neutrophils, activates the CD56^bright^CD16^−^ NK cell subpopulation only via TLR8, highlighting the potential role of TLR8-targeted infiltrating TME-NK cells as a novel tumor immunotherapy [56].

(3) Tumor-associated macrophages

Tumor-associated macrophages are the most extensively infiltrated immune cells in TME [57]. Based on the pro-inflammatory and anti-inflammatory functions of macrophages in response to different stimuli, macrophages are classified into M1 type, which is classically activated, and M2 type, which is alternatively activated. Among them, type M1 is activated by LPS or IFN-γ and plays a pro-inflammatory role, while type M2 is activated by IL-4, IL-13 or IL-10 and plays an anti-inflammatory role. Tumor-associated macrophages typically exhibit an immunosuppressive M2-like phenotype in many kinds of cancers and can promote cancer cell proliferation, immunosuppression, and angiogenesis to support tumor growth and metastasis [58].

The TLR-triggered early immune response plays an important role in polarizing macrophages [59]. Although chemotherapy can significantly reduce the number of MDSCs in breast cancer patients, these MDSCs remain highly T-cell suppressive. And the immunosuppressive mechanisms are STAT3 signaling and increased expression of IDO and IL-10 [60]. TLR1/TLR2 signaling enhances antitumor immunotherapy by promoting the differentiation of monocyte MDSCs into M1-type macrophages to block their inhibitory activity [61]. The chemotherapeutic agent oxaliplatin causes immunosuppression by inhibiting MDSC differentiation into M1-like macrophages. In contrast, the TLR 7/8 agonist R848 reversed oxaliplatin resistance by polarizing MDSCs to the M1 phenotype. the combination of R848 and oxaliplatin resulted in an increase in M1-like macrophages and inhibition of tumor growth, suggesting that the remodeling effect of R848 on MDSCs reversed oxaliplatin-induced immunosuppression [62]. Meanwhile, R848 regulates macrophage migration inhibitory factor (MIF) expression in different cells and organs [63]. TLR9 expression in prostate cancer cells promotes immune evasion through LIF (an IL-6 type cytokine and STAT3 activator) mediated polymorphonuclear MDSC amplification and activation. Therefore, oligonucleotide-based inhibitors (e.g., CpG-STAT3dODN) targeting TLR9/LIF/STAT3 signaling may offer new opportunities for prostate cancer immunotherapy [64].

Tumor-associated macrophages activation inhibits TLR-mediated M1 polarization [65]. Blood-derived tumor-associated macrophages upregulates immunosuppressive cytokines in human gliomas and are associated with significantly lower survival in low-grade gliomas [66]. Resistin, secreted by macrophages in the TME, is mediated through its receptors CAP1 and TLR4 and affects pancreatic cancer progression and chemotherapy resistance [67]. Resistin effect also promotes lung adenocarcinoma metastasis through the TLR4/Src/EGFR/PI3K/NF-κB pathway [68].

Nanoparticles can interrupt these biological interactions within the tumor by altering the tumor-associated macrophage phenotype. Many types of nanoparticles preferentially accumulate in macrophages after systemic administration, and macrophages recognize nanoparticles as foreign bodies and take them up by endocytosis or phagocytosis. The macrophage response to nanoparticles may depend on factors such as dose, route of administration, size, nanoparticle composition, and nanoparticle surface properties [69]. For example, a glucomannan polysaccharide with 1.8 degree acetyl modification (acGM-1.8), a novel PAMP mimetic, specifically activates TLR2, thereby inducing macrophages into an anti-tumor phenotype. In mouse experiments, this polysaccharide has a higher safety profile than previous TLR2 agonists [70]. Targeted M2-like macrophage nanoparticles (PNP@R@MT) efficiently and selectively delivered drugs to M2-like macrophages, M1-like macrophages and DCs. The design takes advantage of the targeting ability of peptide sequence M2pep and delivers R848 to tumor-supporting M2-like macrophages in an efficient and specific manner to achieve reprogramming of M2-like macrophages [71].

(4) T cells

Adoptive cell transfer studies have demonstrated that TLR functions as an important co-stimulatory and regulatory molecule within T cells themselves. By interacting with T cells, TLR agonists reduce the threshold for TCR activation, enhance T cell proliferation and cytokine production, promote memory T cell formation, and alter the suppressive function of Treg cells [72]. Human Treg cells increase glucose consumption and trigger cellular senescence and suppressive effects. TLR8 signaling selectively inhibits glucose uptake and glycolysis in Treg cells, thereby reversing Treg suppression and enhancing in vivo anti-tumor immunity in a melanoma adoptive T cell transfer therapy model [73,74]. TLR agonists are also involved in regulating the motility of Treg cells by upregulating CD69 and CD44, resulting in increased cell activation [75]. TLR2 signaling promotes the antitumor efficacy of CAR-T cell. The Toll/interleukin-1 receptor domain of TLR2 was introduced into CARs targeting CD19 (1928z) and mesothelin (m28z) to generate 1928zT2 and m28zT2. As a result, T cells expressing 1928zT2 or m28zT2 showed enhanced cell proliferation, persistence and effects against CD19^+^ leukemia or mesothelin^+^ solid tumors in vitro and in vivo, respectively [76]. The TLR7/8 agonist imiquimod activates NK cells to kill tumor cells, leading to the release of tumor antigens and the induction of tumor-specific CD4^+^ T cells; at the same time, imiquimod induces the expression of CXCR3, a homologous chemokine receptor on peripheral lymphocytes, which triggers the infiltration and accumulation of CD4^+^ T cells within the tumor and is essential for tumor rejection [77].

(5) Mast cells

Mast cells are unique tissue-resident immune cells in the myeloid lineage. Mast cells exhibit three modes of behavior: (1) direct antigen presentation capacity; (2) regulation of DC migration and effector T cell initiation efficiency; and (3) recruitment of effector T cell subsets to areas of inflammation or infection, and in situ activation of homing T cells to drive an effective inflammatory response [78,79]. Overall, they can change the tumor microenvironment to anti-tumor immunity. In addition, myeloid cell-derived antimicrobial peptide LL-37 promotes lung cancer growth by inducing phosphorylation of protein kinase B and phosphorylation of glycogen synthase kinase 3β mediated by TLR4 in lung tumor cells to activate Wnt/β-linked protein signaling [80]. In other cases, TLR4 signaling can promote proliferation and malignant transformation of hepatic progenitor cells (HPCs) through long-stranded noncoding RNA regulation [81].

## 4. Dual Role of TLRs Activation in the Tumor Microenvironment

TLR activation in immune cells and cancer cells in the TME usually prevents tumor formation and growth. However, excessive or low activation of TLR supports tumor survival and metastasis. The pro- or anti-tumor effects of TLR signaling depend on the specific TLR molecules being stimulated, the cell type with activated signaling, and the downstream signaling cascade in the activated cells [12,82,83] (Summarized in Table 1). TLR3 is commonly expressed in cancer cells. TLR3 recognizes double-stranded RNA and induces activation of NF-κB and IRF3. Activation of NF-κB induces inflammatory cytokines and MMP secretion, which may promote cancer progression, whereas activation of IRF3 induces the production of type I interferon, which leads to apoptosis of cancer cells [84]. TLR3 also promotes tumor elimination or indirectly promotes tumor progression through upregulation of interferon-α/β and NK cell activation [85]. All of the above indicates that TLR3 has a dual role. In addition, for TLR2, in the glioma TME, TLR2 activation in microglia induces downregulation of MHC class II expression in microglia, thereby limiting T cell-dependent antitumor immunity and promoting glioma immune evasion. In contrast, TLR2 induces an increase in MHC I in microglia, contributing to the proliferation and activation of CD8^+^ T cells against brain tumor [86,87]. Similarly, TLR9 has antitumor properties, but inappropriate activation of TLR9 during chronic inflammation may lead to the activation of transcription factors that induce pro-cancer activity. Particularly in virus-induced gynecological cancers and cervical cancers, TLR9 promotes tumor regression by inducing cytotoxic T lymphocytes and reducing the number of MDSC, tumor-associated macrophages, and Tregs. However, TLR9 can also promote tumor progression and enhance the invasiveness of cervical tissue [88].

## 5. Immunotherapy Applications

### 5.1. TLR Agonists and TLR Inhibitors

#### 5.1.1. Functions of TLR Agonists and TLR Inhibitors

TLR agonists trigger the innate immune system and generate antigen-specific T-cell responses against tumors. In addition to these immunostimulatory functions, TLR agonists contribute to the reprogramming of the immunosuppressive TME. Immunomodulatory approaches of TLR agonists can be divided into: (1) molecular modulation by chemical structural modifications: the splicing of key binding components of TLR agonists significantly reduces the immunostimulatory properties of TLR agonists. Molecular coupling of TLR to other therapeutic agents shows a more synergistic immune response than non-coupled equivalent models; and (2) macroscopic modulation by engineered drug delivery systems: efficient delivery of TLR agonists to secondary lymphoid organs rich in immune cells, specific targeting and delivery to immune cells or endosomes, and reduction of toxicity by focused and sustained delivery of TLR agonists [112]. TLR inhibitor development includes antibodies targeting TLRs, TLR-derived transmembrane (TM) peptides, bacterially secreted proteins, and natural or synthetic small molecules, peptides and proteins. Inhibitor development typically targets the extracellular domain of the TLR to block receptor activation. In addition to extracellular structural domains, targeting transmembrane structural domains and Toll/IL-1 receptor structural domains to develop more potent TLR blockers. TLR inhibitors may attenuate TLR-mediated cytokine cascade responses and inhibit overreactive, uncontrolled adaptive immune responses as novel therapies for the treatment or prevention of autoimmune diseases, inflammatory diseases, and cancer [113]. For example, TLR4 inhibitors may prevent inflammation-induced carcinogenesis. However, TLR-4 inhibitors may pose a risk of impaired host immunity [114].

Chemical structure studies suggest that TLR agonists and antagonists share an overlapping binding region. hTLR8 agonists and antagonists have similar structures but have completely opposite biological effects. Hierarchical clustering analysis and molecular docking showed that hydrogen bonding and hydrophobic properties are key features that distinguish hTLR8 agonists from antagonists. Compared with antagonists, agonists have stronger specific hydrogen-bonding properties, while antagonists have stronger non-specific hydrophobic properties [115,116].

#### 5.1.2. Developed TLR Agonists and TLR Inhibitors

A variety of TLR agonists and TLR inhibitors have been developed for use as cancer immunotherapy tools. Some classical TLR agonists have been approved by the FDA for the treatment of cancer patients, while others have been developed for application in preclinical trials to explore the ability of this small molecule modulator to coordinate anti-tumor immunity (Table 2).

TLR agonists approved by the FDA for the treatment in cancer patients include Bacillus Calmette-Guérin (BCG) (activates TLR2, TLR3, TLR4 and TLR9), monophosphoryl lipid (MPL) (TLR4 agonist) and imiquimod (TLR7 agonist) [117,118,119]. In animal models, MPL has been shown to be an effective, non-toxic vaccine adjuvant and can be used as a component of an improved hepatitis B vaccine (Fendrix) [120]. Clinical application of BCG and monophospho lipid A (MPLA) as TLR4 agonists for cancer therapy [121]. Imiquimod is the most commonly used TLR7 ligand in clinical practice and has been approved for the treatment of external genital warts and cancerous skin lesions. After topical application, this TLR7 agonist induces increased production of interferon-α, IL-12, tumor necrosis factor-α, and Th1-susceptible immune responses. Imiquimod enhances the recruitment of myeloid and pDC subtypes and cytotoxic T lymphocytes and increases the ability of antigen-presenting cells to induce reactive T cells [122,123].

In addition, various common TLR agonists include: the TLR2 agonist Pam3CSK4 [124], the TLR3 agonist poly(I:C) [125,126], the TLR4 agonist lipopolysaccharide (LPS) [127] and the TLR7/8 agonist Resiquimod (R848) [128]. The development of TLR9 agonists is described below specifically to further understand the design and application of this small molecule immunomodulator.

In humans, TLR9 is expressed only on the endosomal membrane of B cells and pDC and induces the recruitment of immune effector cells by activating an inflammatory-like innate response and inhibits the abundant presence of MDSC around the tumor mass. Several synthetic CpG oligodeoxynucleotides (ODNs) have been developed as TLR9 agonists. Among them, the first generation CpG ODN mimics molecules with natural CpG motifs; and the second generation CpG ODN has unique modifications to its sequence and structure to form immunomodulatory oligonucleotides (IMO). TLR9 agonists have been accepted for the treatment of hematologic and solid tumors. Specifically, CpG ODN can be used as monotherapy to enhance immune response and induce tumor regression in early clinical trials, but the effect when treating patients with advanced solid tumors was not evident [129,130,131]. In a CT26 subcutaneous mouse tumor model, CpG-A slightly inhibited tumor growth but had no synergistic antitumor effect with the anti-PD-1 antibody; low doses of CpG-B significantly inhibited tumor growth and had a synergistic antitumor effect with the anti-PD-1 antibody; and CpG-C combined with the anti-PD-1 antibody inhibited tumor growth more rapidly and effectively than CpG-B because CpG-B significantly upregulated PD-L1 expression on multiple host immune cells, thereby promoting tumor immune escape [132]. Similarly, another experiment found that Dextran-CpG-B conjugate significantly enhanced lymph node accumulation of CpG in mice and enhanced antitumor immunotherapy [133]. Furthermore, the delivery of TLR9 agonists elicits a T-cell response to low-immunogenic lung tumors. TLR9 agonists contribute to remodeling of the lung TME, leading to the formation of tertiary lymphoid structures near the tumor, CD8^+^ T-cell infiltration of the tumor, DC expansion, and antibody production. And topical delivery of TLR9 agonists enhances the efficacy of immune checkpoint inhibition [134,135]. Chemical coupling of a polyspecific integrin-binding peptide (PIP) with the TLR9 agonist CpG to form PIP-CpG was found to induce tumor regression and enhance therapeutic efficacy with systemic delivery of PIP-CpG compared to non-targeted CpG in invasive mouse models of breast and pancreatic cancer [136]. Notably, TLR agonists exhibit toxicity associated with extensive immune activation after systemic administration. By designing an immune-stimulating antibody conjugates (ISACs), systemic administration of ISAC induces local antigen presenting cells activation in TME while circumventing the typical toxicity associated with systemic delivery of TLR agonists and may serve as a novel therapeutic approach to trigger durable antitumor immunity [137].

**Table 2:** Novel TLR agonists in recent years.

TLR	TLR Agonist	Structural Characteristic	Applications	Combination Therapy	Results	Refs.
TLR7/8	Telratolimod (Tel)	Hydrophobic long chain	Significantly prevent B16F10 or 4T-1 tumor postoperative recurrence and metastasis	Tel@PGE in combination with chemotherapy and radiotherapy	Recruitment of effector CD8^+^ T lymphocytes and the polarization of MDSCs	[138]
TLR7/8	MEDI9197 (3M-052)	Lipophilicity	Intratumoral injection for local TLR7/8 agonism	Combined with T-cell targeted immunotherapy	Th1 polarization, enrichment and activation of NK cells and CD8_+_ T cells	[139]
TLR9	EnanDIM	L-nucleotide-protected	CT26 colon cancer tumor, MC38 colon cancer, B16 melanoma, A20 lymphoma and EMT-6 breast cancer	EnanDIM^®^ Combination Immunotherapy	Secretion of IFN-α and IP-10 and activation of immune cells	[140]
TLR9	MGN1703	DNA-based	Multiple solid tumors	As an alternative to cancer immunotherapy	Activation of innate immune response with DAMP	[141]
TLR9	CMP-001 (vidutolimod)	Virus-like particles	Activate immune cells in peripheral blood	As a novel immunotherapy	Anti-Qβ antibody reaction causes uptake of particles by pDCs, producing IFN-α	[142]
TLR9	IMO-2125	Containing a phosphorothioate backbone	Intratumoral injection	Co-administration of anti-CTLA-4 and anti-PD-1	Create a long-lived tumor-specific immune memory of CT26 antigens	[143]
TLR2	TLR2-L Amplivant (AV)	Splicing of Pam3CSK4 with synthetic long peptide (SLP)	Cancer Vaccination	AV-SLP couples in combination with chemotherapy or photodynamic therapy	Enhance activation of DCs in vitro	[144]
TLR1/TLR2	Diprovocims	With structure-activity (SAR) relationships	As vaccine adjuvant and cancer treatment	Synergistic action with checkpoint inhibitors	Promotes the release of TNF-α from human THP-1 bone marrow cells	[145]
TLR3	ARNAX	Nucleic acid adjuvants	Safe for older cancer patients receiving immunotherapy	As vaccine immunotherapy	Induces tumor-specific memory T cells and durable anti-tumor immunity without inducing systemic inflammation	[146,147,148]
TLR5	Mobilan	Adenovirus	Injected into the prostate of transgenic mice (TRAMP)	Combined with radiation and vaccination	Constitutive activation of NF-κB in vitro and in vivo	[149]

#### 5.1.3. TLR Agonists and Inhibitors in Clinical Trials

Many TLR agonists and inhibitors undergo preclinical studies for their ability to coordinate anti-tumor immunity. The antitumor response is largely attributed to their ability to stimulate antigen presenting cells, which in turn activate tumor-specific T cell responses (Table 3).

### 5.2. Delivery Methods for TLR Agonists and TLR Inhibitors

#### 5.2.1. Nano-Delivery System

The most obvious advantage of nanoparticles is their plasticity, allowing the modification of factors such as particle size, surface charge, composition, hydrophobicity and morphology to optimize the overall immunostimulatory properties. Nanoparticles can be combined with other therapies, including chemotherapy, radiotherapy, and photodynamic therapy. However, nano-delivery systems may not be applicable to all types of tumors due to the heterogeneity of different tumors and individuals [24,169].

Here are some examples to illustrate that nano-delivery system is an important method for TLR modulators. Chemotherapy with TLR 7/8 agonists as adjuvants induced immunogenic cell death (ICD) and in situ vaccination effects, but indoleamine 2,3-dioxygenase (IDO) significantly increased the number of microenvironmental TGF-β, IL-10, MDSCs and Treg cells in tumors, counteracting activated antitumor immunity. The use of an assemblable immune modulating suspension (AIMS) containing an ICD inducer (paclitaxel) and a supra-adjuvant increased cytotoxic T lymphocytes in TME and alleviated IDO-associated immunosuppression. The supra-adjuvant refers to the synergistic combination of R848 and IDO inhibitor (epacadostat) [170]. Mesoporous silica nanoparticles (MSNs) are considered as the most promising inorganic bio-nanomaterials for clinical translation. MSNs combined with anti-PD-1 for cancer therapy, promote cytotoxic T lymphocyte infiltration through TLR4-NFκB axis and lead to in vitro and in vivo Ccl5/Cxcl9/Cxcl10 production to overcome anti-PD-1 resistance, to establish a T-cell inflammatory microenvironment [171]. SBA-15-type Nanoporous silica microparticles (NSiO(2)-MP) were not cytotoxic when the TLR signaling pathway was involved, and NSiO(2)-MP co-treatment did not enhance the secretion of the pro-inflammatory cytokine IL-6 or modulate the secretion of the cytokine IL-10, and did not affect macrophage polarization toward a pro-inflammatory or immunosuppressive state [172]. CD8^+^ T cell-specific nanoparticles are generated by binding anti-CD8a F(ab’)2 fragments to the surface of the particles. Targeted delivery of R848 recruits CD8^+^ T cells into the tumor and also sensitizes the tumor to anti-PD-1 [173]. As an important complement, recent findings show that RNA acts as a molecule that stimulates TLR activation in combination with nano-delivery system. Innate immunostimulation of RNA is induced via endosomal or cytoplasmic pathways that modulate the function of immune cells, tumor cells and immunosuppressive factors as well as stimulate immune cells by recognizing endosomal TLRs to enhance cancer immunotherapy. Nanoparticle-based multifaceted immunomodulatory RNA delivery strategies improve RNA delivery efficiency by improving cellular uptake, RNA stability and accumulation at desired sites (target cells and intracellular compartments) [174]. For different nano-delivery tools, in contrast to other polymeric nanocarriers, nanogels can bind both hydrophilic and hydrophobic drugs in their 3D polymer network by fine-tuning the chemical composition. Nanogel-delivered antigens can induce robust DC activation and T-cell responses at lower antigen doses than soluble antigens [175].

#### 5.2.2. Exosome Delivery System

Tumor cells participate in the shaping of the TME by secreting exosomes. Tumor-derived small extracellular vesicles (SEVs) induce pro-inflammatory cytokine expression and PD-L1 upregulation in M0 macrophages through IL-6/STAT3 and TLR4 signaling pathways, contributing to an immunosuppressive microenvironment [176]. TLR4 activation enhances the immunosuppressive properties of tumor cell-derived exosomes, allowing tumor cells to evade immune surveillance or promote the development of tumor metastasis [177]. Tumor cell-released autophagosomes (TRAPs) convert macrophages to the M2 phenotype, inhibit the proliferation of T cells and promote tumor growth. Autophagosomes-induced macrophage polarization is dependent on TLR4-mediated signaling [178]. Human hepatocellular carcinoma-derived exosome-derived HMGB1 activates B cells and promotes the expansion of TIM-1^+^ Breg cells through TLR2/4 and MAPK signaling pathways, which express high levels of the immunosuppressive cytokine IL-10 and exhibit strong inhibitory activity against CD8^+^ T cells [179]. Gastric cancer cell-derived exosomes induce neutrophil autophagy and induce neutrophil N2 polarization via HMGB1/TLR4/NF-κB signaling to promote gastric cancer cell migration [180]. Heat shock protein 90α (HSP90α) on the surface of autophagosomes stimulates IL-6 production by CD4^+^ T cells via the TLR2-MyD88-NF-κB signaling cascade. IL-6 further promotes IL-10 and IL-21 secretion by CD4^+^ T cells via STAT3. Autophagosomes-induced CD4^+^ T cells in an IL-6- and IL-10-dependent manner suppress CD4^+^ and CD8^+^ effector T cell functions and induce IL-10-producing Breg cells via IL-6, IL-10 and IL-21, thereby promoting tumor growth and metastasis [181]. Exosomal FMR1-AS1 is involved in maintaining the dynamic interconversion balance of cancer stem cell-like cells in female esophageal cancer (ESCC) through the TLR7/NFκB/c-Myc signaling pathway by activating TLR7-NFκB signaling and upregulating c-Myc levels in receptor cells [182]. Epstein–Barr virus-encoded RNAs (EBERs) from extracellular vesicles of nasopharyngeal carcinoma cells promote angiogenesis via TLR3/RIG-I-mediated VCAM-1 expression [183].

Recent studies develop a localized drug delivery system with a step-by-step cell internalization capacity based on hierarchical structural fiber devices, which would be internalized by actively targeting tumor cells to induce ICDs. The TLR7 agonist imiquimod can be released from these particles in the cytoplasm to reprogram M2-like tumor-associated macrophage. Loading the drug into the targeted particles can result in doses of antibodies and small molecules that are less than one-tenth of the standard dose, thereby improving efficacy and possibly mitigate potential toxicity [184].

#### 5.2.3. Liposomal Delivery System

Liposomes are a well-established drug delivery system that enables payload stability and enhanced cellular activity during internalization. Cytoplasmic delivery of antigens can be achieved through surface modification of liposomes by pH-responsive or fusion materials, leading to cross-presentation of exogenous antigens via the “cytosolic pathway”. The introduction of TLR agonists into liposomes enhances their immune-inducing ability [185].

Co-encapsulation of antigenic ovalbumin with TLR9 and STING ligands in liposomes revealed that pH-sensitive liposomes co-encapsulating CpG ODN and cGAMP induced synergistic innate immune responses by increasing type I and type II interferon levels, and that ovalbumin caused specific Th1-biased immune enhancement and induced reversal of the immunosuppressive TME while enhancing effective anti-tumor immune responses [186]. Liposomes targeting the DC immune receptor (DCIR) were supplemented with the immunostimulatory TLR7 agonist TMX-202, and monocytes and mDCs were found to exhibit potent TLR7-specific secretion of the anticancer cytokines IL-12p70, IFN-α 2a, and IFN-γ [187]. Delivery of TLR agonists via complement C3-targeted liposomes activates immune cells and reduces tumor growth. C3 actively binds and wraps foreign particles, allowing them to be taken up into bone marrow cells via complement receptors [188].

### 5.3. Combination Applications for TLR Agonists and TLR Inhibitors

#### 5.3.1. TLR Agonists in Combination with Other Therapies

Radiation therapy is one of the most effective treatments for various solid tumors. Combining novel immune interventions with radiation therapy enhances the expression of tumor-associated antigens, induces immune-mediated tumor stromal targeting, and decreases Treg cell activity. Radiation therapy can also enhance the adaptive immune response to cancer by activating innate immune effectors through TLR-dependent mechanisms [189]. Novel agonists targeting TLR3, TLR7/8 or TLR9 enhance antitumor immunity in combination with radiation therapy. TLR agonists enhance DC-mediated T-cell initiation after radiation therapy and, in some cases, generate systemic antitumor immunity and immune memory cells [190].

The combination of photothermal therapy (PTT) and TLR-mediated immunotherapy triggers antitumor immunity and modulates the immunosuppressive TME. The combination of integrated adjuvant and PTT using nanomaterials allows the production of high-quality anticancer vaccine delivery systems for elderly patients [191].

Combination of TLR9 ligand (CpG) and anti-OX40 antibodies may cure many types of cancer and prevent spontaneous gene-driven cancers. Low doses of CpG injected into tumors induce OX40 expression on CD4^+^ T cells in the TME, while the anti-OX40 antibody triggers a T-cell immune response that is specific for the antigen of the injected tumor [192].

#### 5.3.2. TLR Agonists as Vaccine Adjuvants

Cancer vaccines are used to break immune tolerance and enhance the innate and adaptive immune response to cancer. Cancer vaccines can be cancer cells, cell lysates, proteins, peptides or genetic material encoding certain cancer antigens. Given the poor immunogenicity of the aforementioned tumor antigens, the antitumor activity of such vaccine systems remains unsatisfactory. Therefore, the vaccine system must be enhanced by the incorporation of certain vaccine adjuvants. Due to their strong immunogenicity, TLR agonists have been extensively investigated as vaccine adjuvants to enhance the biological activity of anti-cancer immunotherapies [121]. Immunoadjuvants are molecules that use the tumor as a source of antigen to trigger tumor-specific immune responses. TLR agonists such as CpG, R848, and poly I:C immunoadjuvants interact with TLRs on the DC surface via the endosomal pathway [124,193].

poly-ICLC, a multifunctional immunomodulator for cancer treatment, is a double-stranded RNA complex that consists of poly(I:C) (polyinosinic:polycytidylic acid) stabilized by poly-L-lysine. This synthetic dsRNA mimetic stimulates TLR3 and MDA5, affects IFN-I and IL-15 production, enhances T-cell infiltration into tumor parenchyma, and serves as a strong adjuvant for peptide cancer vaccines [194]. Carbohydrate antigens themselves are usually poorly immunogenic, so they need to be covalently coupled to immunoreactive carrier molecules to be effective, and glycoconjugate vaccines can be formulated using TLR ligands. The two main classes of TLR ligands for conjugate vaccine development include lipopeptides and lipid A derivatives. In particular, monophosphatidyl lipid A and related analogs, which belong to the TLR4 ligands, are capable of triggering T cell-dependent adaptive immune responses [195,196].

#### 5.3.3. Co-Delivery of TLR Agonists/Antagonists with Nanoparticle for Cancer Treatment

Both TLR agonists and antagonists can be used for cancer treatment by enhancing innate immunity, T-cell immunity and cytotoxic antibody function [197]. Given the dual role of TLRs, their activation is fine-tuned during using multiple drug combination methods [198]. Various materials co-deliver different drug combinations for cancer therapy, indicating that rationally designed drug-nanoparticle combinations can enhance anticancer efficacy. Table 4 summarized a series of combination of TLR agonists/antagonists and nanoparticle used for cancer treatment.

## 6. Conclusions

Whether TLRs promote tumor development needs to be observed in the specific TME. Various types of cells in the TME interact with each other through cytokines or chemokines, which in turn lead to changes in the distribution and cell infiltration within the tumor. Both immune cells and tumor cells express TLRs, and the same TLR molecule is expressed in different cells with different roles. Despite these conflicting roles, TLR agonists have been established as anticancer agents that activate immune cells in the TME and promote the expression of cytokines that permit anti-tumor lymphocyte infiltration and inhibit oncogenic signaling pathways [207,208].

This review focuses on the link between TLR activation and the TME mainly through pro-inflammatory mechanisms, where TLR activation affects the level of immune cell infiltration in the TME and consequently the development of tumors. Many TLR agonists have been used in the treatment of malignant tumors, the most important applications being: (1) as vaccine adjuvants; (2) in combination with other therapeutic modalities to achieve optimal efficacy; and (3) in combination with different delivery systems to enhance the ability to target tumor cells.

Nowadays, several clinical trials on TLR agonists and TLR inhibitors are underway, especially in combination with immunotherapy and conventional therapies. As a small molecule modulator, TLR can provide an effective anti-tumor response by activating cytotoxic T lymphocytes, reshaping the tumor immunosuppressive environment, polarizing neutrophils and tumor-associated macrophages, etc. Combination therapy or targeted therapy could better address the serious problems of drug resistance, excessive toxicity and strong immunogenicity. Therefore, development of TLR modulators has a great potential to be an important tool for cancer immunotherapy.

## Figures and Tables

**Table 1:** Different types of TLRs are involved in tumor progression or inhibition through different mechanisms.

Tumors	TLRs	Type	Mechanisms	Refs.
Glioblastoma	TLR4	pro-	TLR4 represses RBBP5 to suppress cancer stem cells (CSCs) properties, but CSCs evade innate immunosuppression by reducing TLR4 expression and downregulating its activity	[89,90]
TGCT	TLR2	pro-	A candidate prognostic factor	[91]
Breast carcinoma	TLR3	pro-	Hypoxia-inducible factor 1α (HIF1α)-dependent angiogenesis	[92]
EAC	TLR4	pro-	TLR4-MyD88-TRAF6-NF-κB	[93,94]
ESCC	TLR4	pro-	Production of immunosuppressive cytokines TNF-α and TGF-β	[95]
HCC	TLR4, TLR7, TLR9	pro-	TLR4-AKT-SOX2 signaling pathway enhances cancer stem cell competence; dysregulated AKT/mTOR/4EBP1 signaling	[96,97,98]
PDA	TLR4	pro-	HMGB-1 and S100A8/S100A9 stimulate TLR4 signaling	[99,100]
NSCLC	TLR4, TLR3	pro-	A candidate prognostic factor	[101,102]
CRC	TLR4	pro-	Cathepsin K (CTSK) mediates TLR4-stimulated polarization of M2 macrophages; TLR4-miR-155 positive feedback loop	[103,104]
DLBCL	TLR9	pro-	Formation of neutrophil extracellular traps (NETs)	[105]
Melanoma	TLR2, TLR4	pro-	Overexpression of MMP2 tends toward a potentially tumorigenic phenotype	[106]
Melanoma	TLR5	anti-	Activation of TLR5 signaling by Salmonella flagella	[107]
TNBC	TLR4	anti-	Infiltration and activation of different immune cell types	[108]
PC	TLR3	anti-	Inhibition of proliferation by mitogen-activated protein kinase and induction of apoptosis by cysteine aspartase	[109,110]
SCCHN	TLR8	anti-	Synergistic activation of NK cell activity by motolimod and NLRP3	[111]
Brain tumor	TLR2	anti-	The glial cell TLR2-MHC I axis contributes to the proliferation and activation of CD8_+_ T cells	[86]

Pro-: pro-tumor; Anti-: anti-tumor; RBBP5: retinoblastoma binding protein 5; TGCT: testicular germ cell tumors; ESCC: esophageal squamous cell carcinoma; EAC: esophageal carcinogenesis; HCC: hepatocellular carcinoma; PDA: Pancreatic ductal adenocarcinoma; NSCLC: Non-Small Cell Lung Cancer; CRC: Colorectal Cancer; DLBCL: Diffuse Large B-cell Lymphoma; TNBC: triple negative breast cancer; PC: prostate cancer; SCCHN: squamous cell carcinoma of the head and neck.

**Table 3:** Completed clinical trials of TLR agonists in multiple combinations with other therapeutic modalities.

Reagents	Phase(s)	Subjects	Results	Refs.
GSK1795091 (TLR4 agonist)	I/II	Health Volunteers	Acceptable safety profile with dose-dependent cytokine and immune cell changes	[150]
NY-ESO-1 vaccine + anti-CTLA4 antibody Ipilimumab (IPI)	I	Patients with unresectable or metastatic melanoma	Enhanced immune infiltration	[151]
CHP-NY-ESO-1 vaccine combined with poly-ICLC	I	Advanced or recurrent esophageal cancer patients	Acquisition of antibody response	[152]
Neoadjuvant Cetuximab and motolimod	Ib	Head and Neck Cancer	Reversal of myeloid-derived suppressor cells (MDSC) inhibition of T cell proliferation	[153]
Vaccination With Poly-ICLC and Peptide-pulsed Dendritic Cells	N/A	Patients with pancreatic cancer	Good tolerance and effective production of antigen-specific T cells	[154]
A transdermal multipeptide melanoma vaccine administered with or without a TLR7 agonist	N/A	Melanoma	TLR7 agonist imiquimod as a vaccine adjuvant	[155]
Topical Imiquimod Plus Nab-paclitaxel	II	Breast Cancer Cutaneous Metastases	Effective in inducing disease regression in refractory breast cancer chest wall metastases, but response is transient	[156]
A long peptide vaccine (LPV7) plus toll-like receptor (TLR) agonists	I/II	Resected high-risk melanoma	Enhanced T-cell response	[157]
Combined Vaccination with NY-ESO-1 Protein, Poly-ICLC, and Montanide	I/II	Patients with High-Risk MelanomaPatients with High-Risk Melanoma	The combination is safe, well tolerated, and induces integrated antibody and CD4^+^ T cell responses	[158]
GNKG168(TLR9 agonist)	I	children with minimal residual disease positive acute leukemia	Induction of an immediate NK response, followed by adaptive T and B cell responses	[159]
TLR8 Agonist, Motolimod (VTX-2337), Combined with Cetuximab	Ib	Patients with Recurrent or Metastatic SCCHN	Motolimod co-administered with cetuximab has safety profile	[160]
IMO-2125 (TLR9 agonist)	I/II	Pancreatic cancer	Triggers immune system response and kills local and distant tumors	[161]
Tilsotolimod (TLR9 agonist)	II/III	Refractory solid tumors and melanoma	Immunomodulation of TME and therapeutic effects on distant tumor lesions	[162]
G100 (TLR4 agonist)	N/A	Patients with Merkel Cell Carcinoma	Induces Antitumor Immune Responses and Tumor Regression	[163]
SD-101 (TLR9 agonist) and Local Low-Dose Radiation	I/II	Untreated Indolent Lymphoma	Combination therapy is safe and well tolerated	[164]
Motolimod with pegylated liposomal doxorubicin	II	Recurrent or persistent ovarian cancer	Combination therapy was well tolerated, but did not significantly improve clinical outcomes	[165]
Peptide vaccine with glucopyranosyl lipid A-stable oil-in-water emulsion	IIA	Patients with resected melanoma	Is well tolerated and leads to an increase in specific T cells	[166]
PF-3512676 (TLR9 agonist)	I/II	Lymphoma	Well-tolerated, induces systemic anti-tumor immunity	[167]
TLR9 agonist combined with radiation	I/II	Mycosis fungoides	Systemic tumor regression	[168]

N/A indicates information that is not available.

**Table 4:** Co-delivery of TLR agonists/antagonists with nanoparticle for cancer treatment.

Combination	Treatment Effect	Refs.
CDNPs	Promote the polarization of tumor-associated macrophages	[199]
R848@NPs+NIR	Effectively internalized by dendritic cells and generate robust anti-tumor memory immunity	[200]
Sunitinib/PD-L1 Blockade/TLR7/8 Agonist-Based Nanovaccine	Reduced MDSC and Tregs, and reduced CD8 T cell depletion	[201]
HPV E7 long peptide/PIC/anti-PD-1 mAb	Treatment of primary drug-resistant tumors	[202]
nMOFs/TLR7 agonist/Anti-CD47 Antibodies	nMOFs are radiosensitizers; the TLR7 agonist imiquimod (IMD) repolarizes immunosuppressive M2 macrophages to immunostimulatory M1 macrophages; and anti-CD47 antibodies block CD47 tumor cell surface markers to promote phagocytosis	[203]
Nanoparticle-Conjugate TLR7/8 Agonist/anti-PD-1 mAb/Flt3L	Leads to efficient activation of DCs in sentinel lymph nodes and promotes proliferation of tumor antigen-specific CD8 T cells	[204]
R848@LNPs	Promote the conversion of tumor-associated macrophages from the M2 phenotype to the M1 phenotype	[205]
RPTDH/R848	Anti-angiogenesis and immune activation	[206]

R848: TLR7/8 agonist; CDNPs: R848-loaded β-cyclodextrin nanoparticles; R848@NPs/+NIR: a combined therapy that involves an immunomodulator (R848)-loaded nanoparticle system and near-infrared light (+NIR); nMOFs: nanoscale metal–organic frameworks; R848@LNPs: peptide-guided resiquimod-loaded lignin nanoparticles; RPTDH: a copper chelating coil-comb block copolymer RGD-PEG-b-PGA-g-(TETA-DTC-PHis).

## Data Availability

Not applicable.
